# Mechanisms of Action of Probiotics in Intestinal Diseases

**DOI:** 10.1100/tsw.2007.26

**Published:** 2006-12-21

**Authors:** Ann M. O'Hara, Fergus Shanahan

**Affiliations:** ^1^ Alimentary Pharmabiotic Centre, University College Cork, National University of Ireland, Ireland; ^2^ Department of Medicine, University College Cork, National University of Ireland, Ireland

**Keywords:** commensal bacteria, enteric infection, inflammation, inflammatory bowel disease, intestinal epithelium, microbiota, pharmabiotic, probiotic

## Abstract

Intestinal microbiota is a positive health asset that exerts a conditioning effect on intestinal homeostasis. Resident bacteria deliver regulatory signals to the epithelium and instruct mucosal immune responses. Recent research has revealed a potential therapeutic role for the manipulation of the microbiota and exploitation of host-microbial signalling pathways in the maintenance of human health and treatment of various mucosal disorders. A variety of pharmabiotic strategies, such as the use of specific members of the microbiota, their surface components, or metabolites, as well as genetically modified commensal bacteria, are being investigated for their ability to enhance the beneficial components of the microbiota. It is clear that engagement with host cells is central to pharmabiotic action, and several strain-specific mechanisms of action have been elucidated. However, the molecular details underpinning these mechanisms remain almost entirely unknown. Understanding how pharmabiotics exert their beneficial effects is critical for the establishment of definitive selection criteria for certain pharmabiotic strategies for specific clinical conditions. Scientifically accredited evidence of efficacy and studies to elucidate the molecular mechanisms of host-microbiota interactions are needed to lend credence to the use of pharmabiotic strategies in clinical medicine.

## INTRODUCTION

The human gastrointestinal tract harbours a diverse bacterial community that comprises more than 1000 different species, and outnumbers human somatic and germ cells tenfold[1]. Historically, microbial research focused on the mechanisms by which enteric pathogens mediate tissue damage and disease. More recently, a circumstantial role of intestinal bacteria in the pathogenesis of various intestinal disorders has been recognised. For example, in genetically susceptible individuals, some components of commensal organisms can trigger aberrant immune responses that contribute to the pathogenesis of inflammatory bowel disease[2]. Innate immune responses to indigenous bacteria prime the immune system and influence adaptive responses to exogenous antigens. It follows that genes that were once survival factors in an earlier era, could now become risk factors for immune hypersensitivity disorders in the modern sanitised environments of developed nations[3]. Gut bacteria have been implicated in obesity and, under certain circumstances, they can fuel the progression towards colorectal malignancy[4,5]. Despite these adverse associations, intestinal microbiota fundamentally impact human health also.

Under normal circumstances, commensal bacteria are an essential health asset that exert a conditioning and protective influence on intestinal structure and homeostasis. Intestinal bacteria protect against infection, and actively exchange developmental and regulatory signals with the host that prime and instruct mucosal immunity[1]. Colonisation of germ-free mice with a single species, *Bacteroides thetaiotaomicron*, has been shown to affect the expression of a variety of host genes. These include genes associated with nutrient uptake, metabolism, angiogenesis, mucosal barrier function, and the development of the enteric nervous system[6]. Interactions between gut-associated lymphoid tissues and colonising bacteria early in life are crucial for appropriate development of functioning mucosal and systemic immunoregulatory systems[7,8]. Thus, individual variations in immunity may be influenced by the composition of the colonising microbiota. Bacterial metabolism confers many benefits to gut physiology, and commensal bacteria represent a rich repository of metabolites that can be mined for therapeutic benefit[1]. Intestinal bacteria are not uniform in their ability to drive mucosal inflammatory responses. Some commensal species such as *B. vulgatus* are proinflammatory[9]. Conversely, other species lack inflammatory capacity, and certain bacteria including strains of bifidobacteria and lactobacilli can even attenuate inflammatory responses[10,11,12].

## PROBIOTICS AS A THERAPEUTIC STRATEGY

At the turn of the last century, the use of "friendly" microbes present in fermented foods for the purpose of health maintenance and disease prevention was first proposed by Metchnikoff[13]. These beliefs have been substantiated by recent research, which indicates that enhancing the beneficial components of the gut microbiota using probiotics represents a realistic therapeutic strategy in the maintenance of human health and in the treatment of various intestinal disorders. A probiotic is usually defined as a live microorganism that, when consumed in adequate quantities, confers a health benefit on the host. However, as our understanding of host-microbial interactions progresses, this definition is continually revised. It is less restrictive to define probiotics as commensal microorganisms that can be harnessed for health benefits.

Criteria for designating a commensal strain as a probiotic include human origin; acid and bile resistance; survival of gastrointestinal transit; nonpathogenic, production of antimicrobial substances; and immune modulatory activity[14]. The most commonly used probiotics include species of lactic acid bacteria (e.g., lactobacilli and bifidobacteria) that lack inflammatory activity. However, other bacteria including nonpathogenic *Escherichia coli*; yeasts, particularly *Saccharomyces boulardii*; and multistrain cocktails, such as VSL#3, have been used as probiotics also. VSL#3 comprises *Lactobacillus casei, L. plantarum, L. acidophilus, L. delbrueckii* subspecies *bulgaricus, Bifidobacterium infantis, B. breve, B. longum,* and *Streptococcus salivarius* subspecies *thermophilus.* Fermented dairy products enriched with probiotic bacteria are a remarkably successful category of functional foods. It is estimated that annual sales of daily-dose probiotic drinks exceed 1.2 billion euro in Europe alone[15].

By definition, probiotics have a high safety profile and the tolerance is usually excellent. Although many of the commercial probiotic products have been officially designated as "generally regarded as safe", some reports of infections probably caused by probiotics have been published[16,17]. However, this is rare, and these isolated incidences have occurred in immunocompromised patients or those with severe underlying disease. Obviously, the administration of probiotics to such patients groups should be approached with caution. Several studies have administered probiotic preparations to children; they are well tolerated and safe[18,19,20].

## THE EVIDENCE FOR PROBIOTIC EFFICACY

Probiotic bacteria have demonstrated health-promoting effects in intervention studies in several clinical conditions. The best evidence for probiotics in any condition is in the treatment and prevention of enteric infections and postantibiotic syndromes. Several meta-analyses studies have established probiotic efficacy in acute infectious diarrhoea and the prevention of antibiotic-associated diarrhoea[21,22,23,24]. Certain probiotics may reduce the recurrence of *Clostridium difficile*–associated diarrhoea also[25,26]. Recently, the Cochrane collaboration conducted a comprehensive systematic review of the evidence for the use of probiotics in infectious diarrhoea in both adults and children. The review concluded that probiotics were a useful adjuvant to rehydration therapy in the treatment of acute infectious diarrhoea[27].

Necrotising enterocolitis is a severe gastrointestinal inflammatory disease that is a common cause of morbidity among premature, low-birth-weight infants. Factors contributing to its pathogenesis include naïve intestinal and immune function, enteral feeding, and gas-producing bacteria. In a number of studies, probiotics have been shown to reduce the incidence and severity of necrotising enterocolitis by contributing to the establishment of a natural, rather than an abnormal, microbiota[28,29]. In affected neonates, probiotics appear to be safe and more effective than other strategies. The administration of probiotics to mothers prior to delivery and to breastfed infants has been shown to influence gut immunity in the newborn positively[30].

Irritable bowel syndrome is a common functional bowel disorder and a role of probiotics in its treatment is promising. The administration of *B. infantis* 35624, but not lactobacilli, has been shown to improve the symptom profile of patients with irritable bowel syndrome[31,32]. In patients with pouchitis, a nonspecific inflammation of the ileal reservoir, probiotic bacteria have demonstrated efficacy in maintaining remission in chronic pouchitis or preventing the development of pouchitis in the first place[33,34,35]. Nevertheless, the wider open clinical experience with probiotics in pouchitis patients is inconsistent and may be related to variability in patient populations or the choice of probiotic preparation. Ulcerative colitis and Crohn's disease, collectively known as inflammatory bowel disease, are chronic relapsing and remitting inflammatory disorders of the gastrointestinal tract. In ulcerative colitis, *E. coli* Nissle 1917, *L. rhamnosus* GG, and VSL#3 have shown efficacy similar to the drug mesalazine in maintaining remission[36,37,38]. Probiotics have induced remission of acute ulcerative colitis also[38,39]. However, in a randomised, double-blind, placebo-controlled trial, *B. infantis* 35624 and *L. salivarius* subspecies *salivarius* UCC118, probiotics that have attenuated disease severity in animal models of colitis, did not demonstrate efficacy in the maintenance of steroid-induced remission of ulcerative colitis[40]. The differences in efficacy between animal and human inflammatory bowel disease may reflect the timing of administration, differences in disease severity, or effective probiotic dose/body weight. *Saccharomyces boulardii* and *E. coli* Nissle 1917 have been effective in the maintenance of remission in patients with Crohn's disease[41,42]. However, controlled studies of probiotics in Crohn's disease did not find efficacy for *L. rhamnosus* GG or *L. johnsonii* LA1 as maintenance therapies for Crohn's disease[43,44]. Larger, well-powered, randomised control trials are needed to determine conclusively whether there is a role for certain strains of probiotics or probiotic combinations in Crohn's disease.

There is evidence to suggest that the gastric colonisation and activity of *Helicobacter pylori* can be inhibited by probiotics. Probiotics do not eliminate the pathogen, but suppress its growth and reduce gastric inflammation[45,46]. In patients with severe acute pancreatitis, *L. plantarum* 299v was protective against pancreatic sepsis[47]. The consumption of probiotics has been linked to the improvement of high cholesterol and lactose intolerance, and the potential therapeutic use of probiotics in the prevention and treatment of human malignancy, atopic/allergic diseases, and rheumatoid arthritis are additional areas of potential application[48,49,50].

## MECHANISMS OF PROBIOTIC ACTION

Experimental models have revealed that probiotics differ greatly in their mechanism of action; any singular mechanism is unlikely to account for all of their clinical effects. Significant differences exist, not only between probiotic species, but also between certain strains. In addition to specific interactions between probiotic bacteria and host immune cells, microbe-microbe interactions confound the complexity of the signalling network *in vivo*. Such complex interactions probably account for the versatility of probiotic action and could explain some of the varying results observed within the different clinical trials. Understanding the various mechanisms of probiotic action is crucial for the establishment of definitive selection criteria for certain strains or combination of strains for specific clinical conditions. Although the molecular details of probiotic mechanisms remain unresolved, numerous studies have indicated that the beneficial effects of probiotics may be either direct or indirect through modification of the local microbiota, epithelial barrier function, intestinal inflammation, or the immune system (Fig. 1).

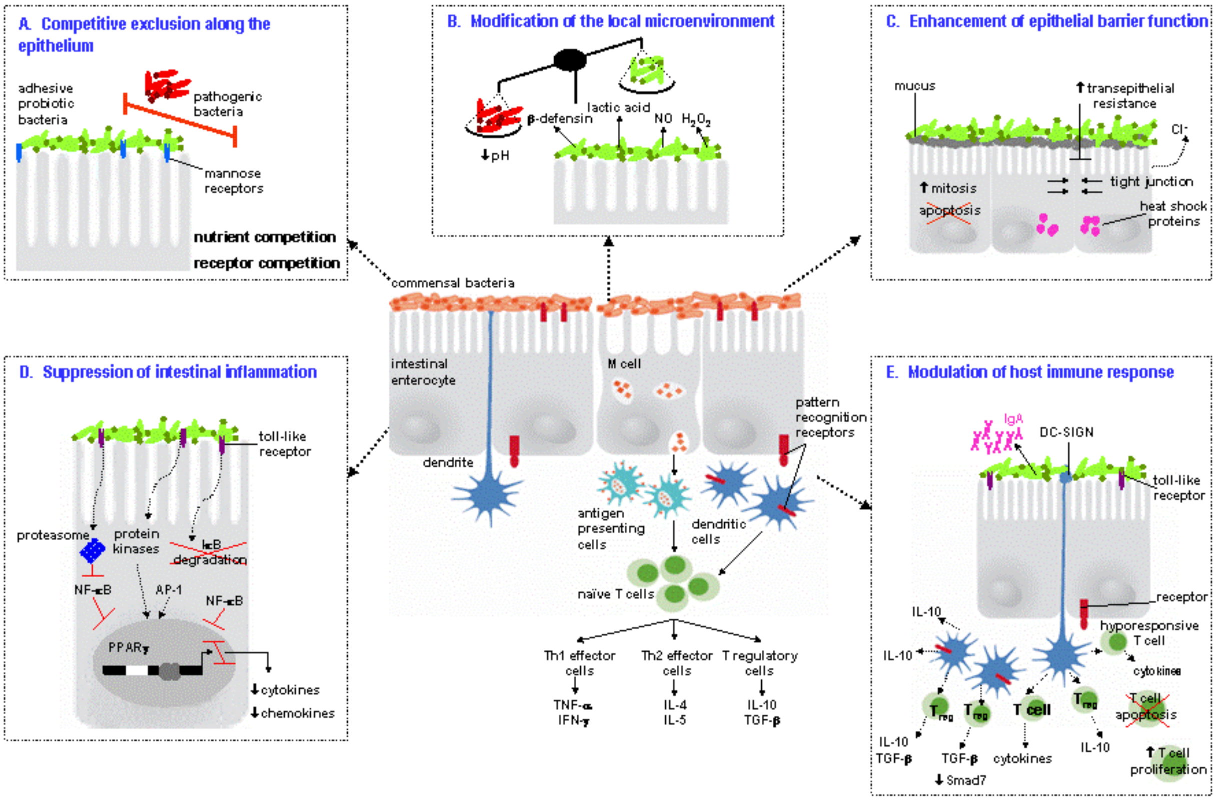

**FIGURE 1.** Mechanisms of action of probiotics in intestinal diseases. In the intestine, immunosensory cells are continually sampling and responding to the microbiota. Pattern recognition receptors expressed by immunocytes mediate the detection of bacterial antigens. Surface enterocytes sense danger signals and secrete immune mediators in response to antigens. Specialised epithelial cells, termed M cells, transport and deliver antigens to antigen-presenting cells, which in turn process antigens and present them to naïve T cells. Dendritic cells also survey and sample the mucosal microenvironment. Dendritic cells act as switches for immune responsiveness and determine the nature of the response by promoting either Th1 or Th2 effector cells or regulatory T cells and their associated cytokines. The figure illustrates several mechanisms of probiotic action that are relevant to intestinal diseases: A, competitive exclusion along the epithelium; B, modification of the local microenvironment; C, enhancement of epithelial barrier function; D, suppression of intestinal inflammation; E, modification of the host immune reponse. These mechanisms are strain-specific and are not mutually exclusive. AP-1, activator protein-1; $Cl^-$, chloride; DC-SIGN, dendritic cell-specific intercellular adhesion molecule 3-grapping nonintegrin; $H_2O_2$, hydrogen peroxide; IFN, interferon; IgA, immunoglobulin A; IL, interleukin; NF-κB, nuclear factor-κB, NO, nitric oxide; PPAR, peroxisome proliferator-activated receptor; TGF, transforming growth factor; TNF, tumour necrosis factor-α; Treg, regulatory T cell.

## Competitive Exclusion Along the Epithelium

The intestinal epithelium is an important barrier that restricts the penetration of luminal antigens and microbes. Interaction between bacterial antigens and host cell receptors is a crucial step in the pathogenesis of many intestinal diseases. Preventing such interactions thus represents a potential therapeutic strategy. Several probiotic bacteria including bifidobacteria and lactobacilli adhere to mucosal tissue in a strain-specific manner[51,52,53,54,55]. This limits nutrient availability to other bacteria, enhances the intestinal persistence of the probiotic bacteria, and limits pathogen access to the epithelium (Fig. 1A).

Genomics-based homology searches have led to the identification of several adhesion factors in probiotic bacteria[56,57]. Surface structures, such as elongation factor Tu and GroEL of *L. johnsonii* LA1, and adhesins from other lactobacilli can bind epithelial cell mucins and mannose[58,59,60,61]. Enteropathogenic *E. coli* are known to bind to epithelial cells via mannose receptors. Therefore, it is feasible that probiotic strains with similar adherence capabilities could inhibit pathogen attachment at these or other binding sites, or impede the penetration of invasive pathogens across the mucosal layer. Moreover, GroEL has been shown to mediate the aggregation of *H. pylori*[59]. Probiotics, such as *L. bulgaricus*, which adhere weakly to the intestinal mucosa, are less effective than adhesive strains against enteric pathogens. Adhesive probiotic bacteria, such as *L. plantarum* 299v, *L. acidophilus* ATCC4356, and *Streptococcus thermophilus* ATCC19258 have been shown to prevent pathogen-induced electrolyte secretion and barrier dysfunction. However, these protective effects were only observed when the probiotics were added prior to pathogen challenge[54,62]. There is evidence to indicate that particular combinations of probiotic strains may have synergistic adhesive effects, thereby increasing the efficacy of a probiotic preparation[63].

In rotavirus infection, *L. casei* DN-114001 has been shown to use soluble probiotic-derived factors to modify the glycosylation state of epithelial cell receptors[64]. This inhibits the adhesion of the virus. In contrast, bacterial species that do not induce glycosylation changes are not protective against the virus. Pathogenic organisms induce diarrhoea by diverse processes; therefore, multiple other mechanisms probably contribute to probiotic-mediated improvement of diarrhoea.

## Modification of the Local Microenvironment

Studies using *in vivo* expression screening technology have identified a variety of probiotic genes that are induced in the murine gastrointestinal tract[65,66]. Such studies indicate that probiotic bacteria are responsive to gut conditions and metabolically active *in vivo*. Administration of probiotic bacteria can modify the composition of the local microenvironment in two key ways. First, probiotic bacteria mediate antimicrobial effects that can directly inhibit pathogenic bacteria; second, they enhance the richness and diversity of the more beneficial components of the gut microbiota (Fig. 1B). Probiotics have been shown to suppress pathogen growth through the release of a variety of antimicrobial factors. These include defensins, bacteriocins, hydrogen peroxide, nitric oxide, and short chain fatty acids, such as lactic and acetic acids, which reduce the pH of the lumen[67].

The effects of the administration of probiotic bacteria on the indigenous mucosa-related microenvironment are poorly understood. Nevertheless, a number of recent studies demonstrate that probiotics play a role in the restoration or maintenance of a protective intestinal microbiota. In patients with pouchitis, VSL#3 therapy increased the diversity of the bacterial community, especially the anaerobic members, whereas the diversity of the fungal flora was repressed[68]. In contrast, patients who relapsed in the placebo group showed reduced microbial diversity. In a randomised, double-blind, placebo-controlled trial, the administration of *L. johnsonii* LA1 to healthy volunteers was found to increase total numbers of bifidobacteria and lactobacilli, as well as faecal lactic acid concentrations. This was coupled with decreased faecal pH and reduced numbers of clostridia[69]. By inhibiting the adverse components and promoting the beneficial components, probiotic bacteria favourably modify the local microbiota.

## Enhancement of Epithelial Barrier Function

Alterations in epithelial transport and barrier functions are a common consequence of a variety of intestinal disorders including enteric infections. Defects in epithelial barrier function may also precede the onset of inflammation in patients with inflammatory bowel disease[70]. In contrast, commensal bacteria help to fortify the epithelial barrier by various mechanisms. For example, colonisation of germ-free mice with the commensal bacteria, *Bacteroides thetaiotaomicron*, induces the expression of the complement-inhibitor, decay-accelerating factor and complement-reactive protein-ductin, a putative receptor for cytoprotective intestinal trefoil factors[71]. Exposure of colonic epithelial cell lines to bacterial ligands also results in apical tightening and sealing of tight junctions and increased transepithelial resistance[72].

Several probiotic bacteria have been shown to preserve epithelial barrier function and prevent and repair mucosal damage triggered by food antigens, drugs (such as aspirin), enteric pathogens, and proinflammatory cytokines[54,73,74,75]. These protective effects are mediated by a number of mechanisms (Table 1). These include the induction of mucin secretion, the maintenance or enhancement of cytoskeletal and tight junction protein phosphorylation, the restoration of chloride secretion, and the augmentation of transepithelial resistance (Fig. 1C). VSL#3 has been shown to up-regulate heat shock proteins known for their ability to maintain cytoskeletal integrity and protect intestinal enterocytes from injury against oxidative stress[76]. Mitogen-activated protein kinases have been implicated in the induction of heat shock proteins by soluble factors from *L. rhamnosus* GG[77]. *Lactobacillus rhamnosus* GG can also exert mitogenic effects by increasing cell proliferation in the villi of germ-free rats, thereby enhancing mucosal regeneration[78]. Moreover, probiotic bacteria can promote cell survival by preventing apoptosis in intestinal epithelial cells through the regulation of both anti- and proapoptotic signal transduction pathways[79]. Together, these varying effects on the epithelium may be instrumental in improving mucosal barrier function and integrity. It is noteworthy that in a study of cytokine-induced barrier dysfunction, a commensal strain, *B. thetaiotaomicron*, was unable to reproduce all of the protective effects mediated by *L. acidophilus* ATCC4356 and *S. thermophilus* ATCC19258. This emphasises that bacteria selected for their probiotic properties may have special abilities that are not necessarily shared by other commensal bacteria[74].

## Suppression of Intestinal Inflammation

Intestinal epithelial cells sense danger signals within the luminal microenvironment. The transcription factor, nuclear factor (NF)-κB, is a master coordinator of immune and inflammatory responses to pathogenic bacteria and other stress signals. However, most commensal bacteria do not activate NF-κB. Instead, some commensal bacteria antagonise NF-κB within enterocytes by a variety of mechanisms. These include degradation of the NF-κB inhibitor IκB-α, or by the nuclear export of the p65 subunit of NF-κB in a peroxisome proliferator-activated receptor (PPAR)γ-dependent manner[80,81]. The anti-inflammatory effects of a number of probiotic bacteria including *Bifidobacterium infantis* 35624 and *L. salivarius* UCC118 have been shown also to be mediated, at least in part, via NF-κB[10] (Fig. 1D). Soluble components from VSL#3 can inhibit IκB degradation by inhibiting epithelial proteasome function[76]. *Lactobacillus reuteri* has been shown to inhibit the nuclear translocation of NF-κB by preventing the degradation of IκB[11]. This was accompanied by an increased expression of nerve growth factor, which has anti-inflammatory properties. This finding implicates a role of the enteric nervous system in host-microbial interactions.

**TABLE 1**
**Probiotic-Mediated Enhancement of Barrier Function in Experimental Models**

| Probiotic | Biologic Effect | Experimental Model | Ref. |
|---|---|---|---|
| *E. coli* Nissle 1917 | Increased transepithelial resistance | T84, HT-29 | [12] |
| *L. acidophilus* | Maintenance of tight junctions and increased occludin expression | Rats | [129] |
| *L. acidophilus* ATCC4356 | Enhanced phosphorylation of actinin and occludin in epithelial cells | HT-29/cl.19A, Caco-2 | [54] |
| | Restoration of chloride secretion and enhanced epithelial resistance | HT-29/cl.19A, Caco-2 | [74] |
| *L. acidophilus* LB | Maintenance of F actin and transport and enzymatic activity | Caco-2/TC7 cells | [130] |
| | Prevented impaired intestinal permeability and protected tight junction proteins | HT-29 | [73] |
| *L. acidophilus* R0011 | Improved intestinal barrier function | Rats | [131] |
| *L. brevis* | Reduced colonic permeability | Rat | [132] |
| *L. casei* | Enhanced epithelial cell glycosylation | HT-29/MTX cells | [64] |
| *L. casei* GG | Up-regulation of MUC2 expression | Caco-2 | [133] |
| *L. helveticus* R0052 | Improved intestinal barrier function | Rats | [131] |
| *L. plantarum* 299v | Up-regulation of MUC2 and MUC3 mRNA and enhanced mucin secretion | HT-29 | [52,134] |
| *L. rhamnosus* GG | Up-regulation of MUC2 and MUC3 mRNA and enhanced mucin secretion | HT-29 | [52,134] |
| | Promoted cell proliferation in the villi | Germ-free rats | [78] |
| | Prevented apoptosis by activating signal transduction pathways in intestinal epithelial cells | YAMC cells, HT-29 | [79] |
| | Induction of cytoprotective heat shock proteins by activating signal transduction pathways in intestinal epithelial cells | YAMC cells | [77] |
| *S. thermophilus* ATCC19258 | Enhanced phosphorylation of actinin and occludin in epithelial cells | HT-29/cl.19A, Caco-2 | [54] |
| | Restoration of chloride secretion and enhanced epithelial resistance | HT-29/cl.19A, Caco-2 | [74] |
| VSL#3 | Enhanced epithelial resistance | IL-10 deficient mice; T84 | [97] |
| | Increased transepithelial resistance and increased mucin expression | T84, HT-29 | [12] |
| | Induction of cytoprotective heat shock proteins | YAMC cells | [76] |

NF-κB transcriptionally regulates interleukin (IL)-8, a potent neutrophil-recruiting and activating chemokine. IL-8 is secreted by intestinal enterocytes in response to several pathogenic bacteria[82]. A variety of probiotic bacteria including VSL#3, *L. reuteri*, *L. salivarius* UCC118, and *B. infantis* 35624 have been shown to suppress IL-8 secretion from infected intestinal epithelial cells[10,11,12]. Moreover, probiotic treatment limits inflammatory cytokine secretion from inflamed mucosal explants from inflammatory bowel disease patients[83,84]. Although the NF-κB pathway has been most frequently implicated, other intracellular signal transduction pathways have also been associated with the protective effects mediated by various probiotic bacteria. These include mitogen-activated protein kinase, protein kinase B, activator protein-1, and PPAR-γ pathways[12,79,85,86]. Many of the mechanisms are strain specific and other signal transduction pathways are likely to account for the anti-inflammatory effects of other probiotic bacteria.

## Modulation of Host Immune Response

Commensal bacteria can modulate the immune system at both a systemic and local level. Signals mediated by commensal bacteria or their ligands are essential for optimal mucosal and immune development, and to maintain and repair the gut[87,88]. In the intestine, immunosensory cells, such as enterocytes, M cells, and dendritic cells, are continually sampling and responding to intestinal bacteria[1]. These immunocytes express pattern recognition receptors including toll-like receptors (TLRs) that engage bacterial signals, such as lipopolysaccharide, lipotechoic acid, bacterial DNA, and flagellin. This contributes to the activation of transcription factors and proinflammatory cascades in immunosensory cells. TLRs play a central role in the interpretation of the microenvironment and the discrimination of pathogen from commensal. Oral consumption of probiotics is associated with immune engagement and demonstrable systemic immunologic changes[89]. It appears that probiotics serve to mimic the commensal microbiota and exploit host-microbial signalling pathways (Fig. 1E). Of note, immune stimulation by probiotic bacteria in the gut can enhance immune protection at distal mucosal sites such as the urogenital and respiratory tracts[90].

Dendritic cells (DCs) sample bacteria and prime adaptive immunity by shaping T-cell responses and regulating the balance of T helper (Th) cell and regulatory T-cell responses in the intestinal mucosa. Different strains of lactobacilli and other probiotic bacteria can modulate DC function by differentially inducing their maturation and the expression of cytokines, such as the regulatory cytokine IL-10[91,92]. It is interesting to note that DCs from different lymphoid compartments exhibit divergent cytokine responses to probiotic and pathogenic bacteria[93]. DC-lactobacilli interactions appear to be mediated, at least in part, by the binding of lactobacilli to a pattern recognition receptor termed DC-SIGN (DC-specific intercellular adhesion molecule 3-grabbing nonintegrin)[94]. On the other hand, lactobacilli that did not interact with DC-SIGN failed to induce IL-10–producing regulatory T cells. In animal models of disease, the therapeutic effects of probiotics are associated with a reduction in inflammatory cytokines, such as tumour necrosis factor-α and interferon-γ, and an induction of regulatory cytokines, particularly transforming growth factor (TGF)-β[89,95,96,97]. In certain clinical conditions, the protective effects of some probiotic bacteria, such as *B. infantis* 35624, have been associated with a normalisation of cytokine imbalances[31].

Some strains of probiotic bacteria, such as *L. casei* or *L. reuteri*, but not *L. plantarum*, can promote tolerance-inducing DCs by priming DCs to drive the development of regulatory T cells[94,98]. These regulatory T cells produce high levels of IL-10 and suppress the proliferation of effector T cells in an IL-10–dependent manner. Similarly, VSL#3 can ameliorate Th1 cell-mediated murine colitis by restoring cytokine balance through the induction of IL-10- and TGF-β-bearing regulatory T cells[99]. Increased TGF-β signalling induced by *B. breve* in preterm infants has been associated with reduced expression of Smad7, a negative regulator of TGF-β[100]. Interactions between DCs and *L. rhamnosus* GG can induce hyporesponsive T helper cells[101]. Furthermore, probiotic bacteria may facilitate the polarisation of the naïve immune system by skewing it from Th2 to Th1 responses, thereby promoting humoral and cell-mediated immunity[102]. The promotion of Th1 immune responses by probiotic bacteria may account for their reported suppression of symptoms of atopic diseases, which are typically driven by skewed Th2 responses[98]. Probiotics that suppress that production of anti-inflammatory cytokines and/or enhance the production of IL-10 and TGF-β confer protection against atopic diseases in infants[103].

Proliferation of T cells in response to antigen stimulation is required to expand the T-cell pool and generate functional effector cells. It has been proposed that *E. coli* Nissle 1917 limits intestinal inflammation by attenuating the expansion of newly recruited T cells into the mucosa[104]. Probiotic bacteria also impact on T-cell apoptosis in a species-specific manner. *Escherichia coli* Nissle 1917 does not induce T-cell apoptosis, whereas the apoptotic abilities of *L. brevis* CD2 have been associated with probiotic arginine deiminase and/or sphingomyelinase activity[104,105].

Increased levels of secretory immunoglobulin (Ig)-A and numbers of phagocytic Kupffer cells were recorded in germ-free mice monoassociated with the probiotic *Saccharomyces boulardii* compared with germ-free controls[106]. This comparative study suggested that in addition to enhancing cell-mediated immune responses, probiotic bacteria could also augment innate and humoral immune responses. In agreement with other studies, a recent randomised, double-blind, placebo-controlled trial demonstrated that the administration of two probiotic bacteria, *L. gasseri* CECT5714 and *L. coryniformis*, increased the proportion and activity of phagocytic and natural killer cells as well as levels of IgA in healthy adults[107,108,109]. The administration of probiotic bacteria in infectious challenges leads to an increase in the levels of pathogen-specific IgA [90], and IgA responses are enhanced in formula-fed infants supplemented with probiotics compared with infants receiving placebo[110]. Of note, the induction of IgA in the gut is heavily dependent on TGF-β, which is also closely involved in the maturation of regulatory T cells[111]. It has been postulated that probiotic strains that are capable of increasing antibody production in the gut may have potential as adjunct therapies to boost immune responsiveness to oral vaccination[112].

## THE CASE FOR PHARMABIOTIC ACTION

Direct interactions with viable bacteria are required for the protective effects mediated by certain strains of probiotic bacteria[11,54]. Nonetheless, whether or not the beneficial actions of all probiotic strains are dependent on live microorganisms or whether oral administration is required for clinical efficacy is uncertain. The answers to these questions are probably multifactorial and strain specific.

In mice, the subcutaneous administration of *L. salivarius* UCC118 was shown to attenuate colitis and proinflammatory cytokine production and protect against collagen arthritis[48]. This raises the possibility that probiotic bacteria might not have to be taken orally to have therapeutic benefit. Moreover, nonviable irradiated probiotic bacteria and the subcutaneous administration of DNA derived from VSL#3 have demonstrated protective effects in a number of animal models of colitis[113]. These effects were shown to be mediated not by bacterial metabolites or ability to colonise the colon, but by probiotic DNA binding to TLR9. Bacterial DNA contains immunostimulatory sequences which engage the host TLR9 receptor. These sequences, as well as some of their synthetic oligonucleotides, have also demonstrated protective effects in murine models of colitis and in biopsies from patients with active ulcerative colitis[114,115]. Another study demonstrated that DNA from VSL#3, but not *E. coli*, attenuated NF-κB signalling and inhibited proinflammatory cytokine secretion in intestinal enterocytes[116].

Secreted bioactive molecules or surface proteins of probiotic bacteria may also modulate host immune responses. Irradiated or sonicated probiotic bacteria can affect the maturation and cytokine secretion profile of DCs[91,92]. Supernatant from cultures of *B. breve* C50 can activate DCs via a mechanism that involves the peptidoglycan receptor TLR2[117]. Metabolites derived from *B. breve* C50 and *Streptococcus thermophilus* 065 have demonstrated anti-inflammatory effects that are not lost by crossing the epithelial barrier[118]. The composition of cell surface structures, such as lipotechoic acid of *L. plantarum* NCIMB8826, have been shown to differentially impact on the immune system through a mechanism that involves TLR2[119]. A recent report demonstrated that administration of a high exopolysaccharide-producing lactobacillus (*L. delbrueckii* subspecies *bulgaricus* B3) attenuated experimental colitis significantly more than a low exopolysaccharide-producing strain (*L. delbrueckii* subspecies *bulgaricus* A13)[120]. Conjugated linoleic acid produced by some probiotic bacteria also has important anti-inflammatory properties[121]. Collectively, these reports challenge the traditional assumption that live bacteria are required for therapeutic efficacy. As a result, the less-restrictive term "pharmabiotic" has been coined to encompass all forms of microbial manipulation in therapeutics[1]. Pharmabiotics comprise probiotics, as well as prebiotics and synbiotics, live and dead organisms, components and metabolites thereof, and genetically modified commensal bacteria.

## DESIGNER PROBIOTICS

The genetic modification of bacteria for the site-specific delivery of therapeutic molecules represents a realistic pharmabiotic strategy. In mice, genetically engineered *L. lactis* has been used to deliver IL-10 or cytoprotective trefoil factors locally to the gut[122,123,124]. More recently, in the first human trial with genetically engineered therapeutic bacteria, ten Crohn's disease patients were treated with modified *L. lactis* in which the thymidylate synthase gene was replaced with a synthetic sequence encoding human IL-10. When the modified bacteria are deprived of thymine or thymidine, they are not viable. Neither thymine nor thymidine is readily available in the external environment, thereby limiting the viability of the excreted organism. The treatment was safe, disease activity was reduced, and the modified bacteria were biologically contained[125]. Therefore, bacterial-based topical delivery of biologically active proteins represents a highly promising and safe therapeutic strategy for combating mucosal diseases. The results of larger placebo-controlled trials with this modified *L. lactis* are eagerly awaited.

Recombinant "designer" probiotics that express molecular mimics of host toxin receptors on their surface are being investigated for their ability to bind bacterial toxins, thereby preventing enteric infections[126]. In a recent study, a chimeric lipopolysaccharide containing a glycosylated lipid that mimics the cholera toxin receptor was expressed into nonpathogenic *E. coli* CWG308[127]. The recombinant probiotic could bind cholera toxin, inhibit its cytotoxicity, and also protect infant mice from challenge with *Vibro cholerae.* Whether this or similar recombinant probiotic strategies are efficacious in enteric infections in humans has not yet been demonstrated. Nonetheless, the potential for these designer probiotics is limited only by one's imagination, but public health and other safety concerns must be resolved before routine clinical use in humans.

## CONCLUSIONS

The increasing availability of commensal genomes should facilitate the identification of commensal effector molecules or other components with pharmabiotic potential[128]. The possibility of using these molecules to target distinct points of intracellular signalling cascades specifically might alleviate inflammation in a target area and overcome the global immunosuppressive effects associated with current therapies. By combining comparative genomic-based approaches with molecular models, it should become possible to select a particular probiotic or pharmabiotic strategy for a specific benefit. Overall, the rationale of pharmabiotic therapy appears to be justified. However, clinical evidence of efficacy requires validation, and unsubstantiated health claims need more stringent regulation. These issues can be resolved through larger, rigorously designed, placebo-controlled, double-blind clinical trials. The microbial, immunological, and functional characteristics of individual probiotic strains and their effects in different clinical conditions require clarification. Moreover, in order for consumers and clinicians to endorse pharmabiotic strategies unequivocally, it is crucial to identify the precise mechanisms by which probiotics and pharmabiotics influence human health. Host-microbial signalling is central to pharmabiotic action, and although the various modes of action described here are multifactorial and strain specific, it is important to consider that they are not mutually exclusive. Furthermore, the molecular details behind these mechanisms remain almost entirely unknown. Further studies of physiological interactions within the complex network of host-microbiota and microbial-microbial signalling in gut health and disease should lead to the optimal exploitation of pharmabiotic approaches for different clinical conditions.

## ACKNOWLEDGEMENTS

The authors are supported in part by Science Foundation Ireland. Professor Shanahan has been affiliated with a university campus-based company (Alimentary Health Ltd). The content of this article was neither influenced nor constrained by this fact.
